# Words and Language Matter: Improving Older Person's Healthcare Outcomes Through Use of Age‐Positive Language

**DOI:** 10.1111/1742-6723.70295

**Published:** 2026-06-11

**Authors:** Nemat Alsaba, Carolyn Hullick, Rebecca Heath, Denise Hobson, Bridie Mulholland, Ellen Burkett

**Affiliations:** ^1^ Gold Coast Hospital and Health Service Gold Coast Queensland Australia; ^2^ Faculty of Health Science and Medicine Bond University Gold Coast Queensland Australia; ^3^ Hunter New England Health Newcastle New South Wales Australia; ^4^ University of Newcastle Newcastle New South Wales Australia; ^5^ Australian Commission on Safety and Quality in Health Care Sydney New South Wales Australia; ^6^ Royal Hobart Hospital Hobart Tasmania Australia; ^7^ The University of Queensland Brisbane Australia; ^8^ Princess Alexandra Hospital Brisbane Australia

**Keywords:** advocacy, ageism, cognitive bias, communication, healthcare outcome, older adult, older person, person‐centred care, respect, words

## Abstract

Ageism is a public health emergency affecting both the health outcomes of older people and the global economy. Emergency Departments are environments where emergency clinicians often face increased cognitive load and are more susceptible to cognitive bias, which can influence patient outcomes. With an ageing population, sustainable healthcare requires a multifaceted approach that includes awareness of the extensive harms of ageism. The language and words used by healthcare providers both directly and indirectly impact patient experience and health outcomes. Consciously transitioning to age‐positive language is one strategy emergency clinicians can use to combat ageism and improve healthcare experiences and outcomes for older persons presenting for emergency care.

## 1 Background

Preparing and upskilling healthcare professionals (HCPs) to meet the needs of the rapidly ageing population is imperative to ensure sustainability of our healthcare system. Ensuring care provided to older people is respectful, high‐quality, and person‐centred requires HCPs to choose words and language that are age‐positive.

### 1.1 Words and Language

Language encompasses both verbal communication (including spoken and written words) and gestural or nonverbal communication (including body language, facial expressions and paralinguistic cues such as tone of voice, speech rate and volume).

The words and language we employ significantly influence our thoughts and emotions, which in turn shape our behaviours and attitudes. This principle applies equally within the realm of healthcare, including the emergency department (ED). The words and language used in healthcare influence HCPs using them, shaping their perception, which in turn influences their behaviours, attitudes and clinical decisions (or omissions). Ultimately, this can impact the broader team's attitude, influence the medical care and treatments offered, and impact patient health outcomes [1, 2, 3].

Words also significantly influence their recipients, impacting patients' perceptions of the care they receive, as well as their self‐worth and perceived value. Additionally, words play a pivotal role in shaping therapeutic relationships [4], and patients' responses to medical interventions and treatments, ultimately affecting their health outcomes [5, 6].

### 1.2 Language and Cultural Context

The meanings of words are relative to the cultural contexts of the persons using and receiving them. Cultural differences influence the preferred terminology used in healthcare communication, and what is considered offensive in one language or culture may be respectful in another [7]. For example, ‘seniors’, which older people may interpret as pejorative, in some South Asian countries, when translated into the local language, implies respect and recognised experience [7].

Understanding and respecting the older person's cultural background helps HCPs choose words and communication styles that align with the older person's preferences, thereby enhancing engagement and potentially improving healthcare outcomes. Furthermore, language preferences are shaped not only by cultural contexts but also evolve over time; we can only understand and respond to these changes through awareness, reflection and research.

### 1.3 Implicit Bias and Ageism

Implicit bias pervades the healthcare system, influencing patient care, treatment options and negatively impacting health outcomes [8]. Implicit bias among HCPs affects patient‐provider communication and interaction, leading to disparities in treatment recommendations, such as pain management, and patient adherence to treatment [9]. HCPs under increased cognitive load, such as emergency clinicians, are more prone to such bias [10, 11].

Ageism is ubiquitous [12]. The financial burden of ageism on global healthcare expenditures is estimated at $63 billion USD in 1 year [13]. Its detrimental impact hinders effective healthcare delivery for older people [12, 14, 15].

Despite growing awareness of the importance of effective communication between HCPs and patients in fostering a positive healthcare relationship, the use of pejorative diagnostic terms such as ‘social admission’ and ageist alienating language remains widespread in healthcare, including in ED. Whilst much of the evidence we reference has been derived from environments other than the ED, patients' preferences are likely to be common across different parts of the healthcare system.

We call on all emergency clinicians to eliminate age‐negative language and pejorative diagnostic labels.

## 2 Existing Descriptors of Older Persons and Preferred Terminology

**FIGURE 1:**
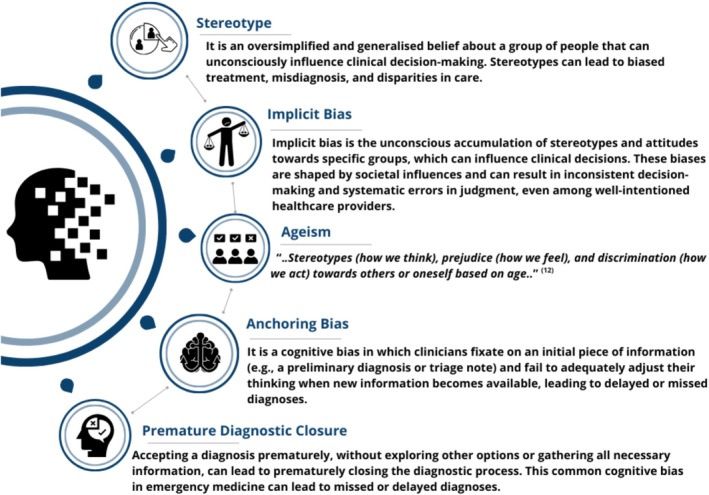
Types of cognitive biases that can negatively impact the care of older persons.

Choosing carefully the words and language we use in healthcare is about providing respectful, dignified, person‐centred care to improve the healthcare outcomes for older people. In this article, we examine the compilation of:

- existing ‘*words*’ or descriptors that fall under the category of ‘age‐negative’ or ‘ageist’ language, pertaining to older persons in healthcare, and
- words that may result in adverse healthcare outcomes through implicit bias, anchoring bias, premature diagnostic closure or ageism (Figure 1).

### 2.1 Age‐Negative Language

#### 2.1.1 Population Descriptor

Older people constitute a diverse, not homogenous, group. Although frailty prevalence increases with age, ageing is not synonymous with frailty or cognitive decline [7]. Describing the older population with terms that align with their expressed preferences is key to respectful engagement. Terms such as ‘elderly’, ‘old’, and ‘senior’ are commonplace in medical literature and parlance. However, such words are identified in the literature and via consensus [7] as being ageist and are often perceived by older people as pejorative and demeaning, portraying dependency and fuelling negative stereotypes used to undermine and undervalue older people [12, 16, 17, 18].

Another age‐negative term used to describe older people is ‘geri‐’ or geriatric patient. Geriatric Medicine, the medical specialty focused on providing care for the unique health needs of older persons, should not be used to describe a person or a cohort.

In alignment with the 2021 guide published by the National Ageing Research Institute (NARI) [19] and the 2022 International Consensus Statement on Reframing Ageing, it is recommended that clinicians use the terms ‘older person’ or ‘older people’ [7].

It must be noted that there is an important distinction between ***Elder*** used in Indigenous Australian culture and the word elderly. In Indigenous culture, ***Elder*** signifies utmost respect and refers to a person recognised and trusted by their community as a custodian of cultural knowledge, and for their wisdom and contributions to community care services, without being linked to reaching a certain age [20].

#### 2.1.2 Infantilising or Patronising Language

Shaw et al. [21] define elderspeak as ‘… *a form of communication overaccommodation used with older adults that is evidenced by inappropriately juvenile lexical choices and/or exaggerated prosody; arises from implicit ageist stereotypes*’. Elderspeak is commonly observed in healthcare [21], where HCPs use oversimplified linguistic *childish and diminutive terms* and paralinguistics, with *raised pitch and overly exaggerated pronunciation* styles that resemble baby talk, often including inappropriate terms such as ‘dear,’ ‘dearie,’ and ‘sweetie’ when communicating with older persons [21, 22]. Other forms of elderspeak include tag questions, which are added at the end of a statement; they do not invite responses but instead create the illusion of choice, for example, ‘You don't want CPR? Do you?’

While elderspeak may stem from a genuine desire to show care, it can also arise from a need to exert control. This might lead to negative self‐perceptions in an older person, including expressions of frustration or unmet needs in people living with dementia [21]. Avoiding elderspeak and using respectful person‐centred language leads to more successful communication and serves as an effective non‐pharmacological way to meet patients' needs, reduce resistive behaviour and improve the quality of dementia care [22, 23].

We recommend avoiding any form of elderspeak, patronising language, and replacing it with respectful, person‐centred language. A crucial step in avoiding elderspeak is recognising its presence in all its linguistic and paralinguistic forms and its impact on older people.

#### 2.1.3 Disease or Deficit‐Centred Versus Person‐Centred Language

The older person's lifetime of experiences and contributions are often diminished to disease‐ or deficit‐centred terms such as ‘*demented*’, ‘*frail*’ or ‘*delirious*’. These terms are pejorative and reinforce stigma, as they equate people and their identity with illness [7, 24], which can negatively impact the healthcare outcomes of older people with these conditions. In alignment with previously published recommendations [7, 19], we advocate use of person‐centred language, such as ‘person living with dementia’.

#### 2.1.4 Negatively Framed Language

***Silver Tsunami*** is a phrase commonly used in scientific literature and by health administrators to describe population ageing. Such negative framing may be perceived as erroneously shifting the blame for lack of preparedness of the health system and health administrators for population ageing onto older people.

We support previous calls to use facts associated with neutral or positive descriptors, such as ‘ageing population’ or ‘demographic changes’ [7].

A summary of the age‐negative language discussed in this section and its impact, paired with alternative age‐positive language, is presented in Table 1.

**TABLE 1:** Age‐negative language and suggested age‐positive alternatives.

Category	Age‐negative language	Age‐positive alternative
Population descriptor	Elderly	**Older person**
	Old	**Older adult**
	Seniors	
	Geri patient	
Infantilising or patronising language	Elderspeak	**Engage in respectful, person‐centred communication**
	‘Dearie’ and ‘sweetie’	**Address the older person with their name or preferred descriptor/prefix**
	‘*You don't want to get an injection? Do you?*’	**Avoid Tag questions**
Disease or deficit‐centred versus person‐centred language	Demented	**Person living with dementia**
	Frail	**Person living with frailty**
	Delirious	**The diagnosis is delirium**. *Mr Lee has delirium*. *Mrs Smith's diagnosis is delirium*.
Negatively framed language	Silver tsunami	**Population ageing**

### 2.2 Words That May Result in Adverse Healthcare Outcomes Through Implicit Bias, Anchoring Bias, Premature Diagnostic Closure, or Ageism

Cognitive biases are common in healthcare. ED clinicians, often managing critically unwell patients requiring rapid decision making, rely heavily on system 1 thinking, which is fast, intuitive, heuristic and unconscious. System 1 thinking is more susceptible to the impacts of emotional valence and cognitive bias [25]. These biases may negatively influence medical decision‐making, sometimes resulting in serious and unfavourable outcomes. Shedding light on words and phrases that impact the outcomes of older people through ageism, stereotypes, and cognitive bias, including premature diagnostic closure, implicit and anchoring bias, is an attempt to discover some of the blind spots in our communications.

#### 2.2.1 Poor Historian

The term ‘poor historian’ is sometimes used to describe an older person who is unable to give a succinct and detailed history of their health issues. Use of this terminology may shift the responsibility of obtaining a clear history onto the patient [26], when in reality, it may highlight the HCP's lack of effort to adequately assess for cognitive impairment, delirium or communication challenges and to seek collateral history from caregivers, general practitioners or paramedics.

Documentation of a vague or poor historian in medical records is associated with a 3.1‐fold increased likelihood of a final diagnosis of cognitive impairment, delirium or both [27]. This highlights anchoring bias and potential lack of consideration among HCPs regarding the reasons behind the difficulty in obtaining history, such as new acute cognitive impairment (delirium), undiagnosed chronic cognitive impairment (dementia) or communication challenges such as hearing and speech challenges. Understanding and reframing the challenge of a complex history should include considering the cause [26] and in some cases it might necessitate the utilisation of cognitive screening tools.

We advocate that clinical notes should include an objective description of a person's ability to provide information: for example, ‘Patient cannot recall their medications’. If an older person's history is unclear, this should trigger an assessment for an underlying cause and prompt the clinician to seek collateral history.

#### 2.2.2 Pleasantly Confused

Confusion in an older person can be acute, chronic or acute on chronic and may be caused by either delirium, dementia or delirium superimposed on dementia. While dementia is described as ‘… an insidious neurodegenerative condition, is characterised by chronic and progressive cognitive decline…’ [28], delirium is known as acute brain failure, resulting in acute change in mental status that is reversible and often triggered by acute illness, surgery, injuries or adverse effects of medications [29], which requires urgent recognition and response. Delirium is also one of the most common and significant complications that an older person may face when admitted to hospital [30]. Unfortunately, delirium is frequently overlooked and often not taken seriously [31]. When a diagnosis of delirium is missed in ED, 6‐month mortality increases to a clinically and statistically significant degree [32].

The term ‘patient is pleasantly confused’ may refer to a patient experiencing confusion who might have delirium or dementia [33]. The phrase ‘pleasantly confused’ can downplay the seriousness of delirium and frame it negatively. Rather than rendering an accurate diagnosis, we misrepresent delirium as ‘pleasant’. Patients consistently report delirium as an unpleasant and often distressing experience, even if it is not associated with agitated behaviour [34, 35, 36].

When looked at from another angle, clinicians might unconsciously use this term to project their own feelings of relief when caring for an older person with hypoactive ‘quiet’ delirium, in contrast to their experiences with hyperactive ‘restless and agitated’ delirium, which can be challenging and far from pleasant for clinicians. The use of this term also affects outcomes for older patients, as it implies the condition is not serious which is far from true, as delirium predicts death, increased length of stay, functional and cognitive decline including dementia, post discharge depression, lower quality of life and an increased likelihood of discharge into a residential aged care facility [37]. Notably, hypoactive delirium often has a worse prognosis, potentially due to its tendency to be underrecognised [38]. Any older person with confusion should be considered to have delirium until there is supporting evidence of dementia, with the absence of any acute cognitive deterioration [39].

We recommend eliminating the term ‘pleasantly confused’ from the medical lexicon and replacing it with an objective description of the patient's behaviour, such as ‘patient lying in bed with no agitation’, to facilitate the assessment of behavioural changes over time. We also recommend using precise language and consistent terminology when describing the confusional state [30, 33]. For instance, ‘patient has delirium’ or ‘patient living with dementia’.

#### 2.2.3 Mechanical Fall

Globally and in Australia, falls are the leading cause of trauma‐related death and disability [40, 41]. Falls from standing height are the most common reason for older people to visit ED [42] and account for the largest majority of Injury Severity Score (ISS) greater than 15 [43]. Falls are often caused by multifactorial contributors (extrinsic, intrinsic and situational) and require comprehensive assessment. It is important to move beyond the term ‘mechanical fall’ as this misnomer can lead to bypassing critical thinking and may result in anchoring bias [44], leading to a less thorough assessment of underlying intrinsic contributors and may miss the opportunity to consider remediable contributors. This is highlighted by an audit comparing ‘mechanical falls’ and ‘non‐mechanical falls’, which found no difference in ED presentations, subsequent falls, hospital admissions, or death rates between falls labelled as mechanical versus non‐mechanical [45].

We advocate applying advanced, structured trauma care for falls from standing height and performing thorough assessments to identify the causes of falls. This approach helps avoid premature diagnostic closure and fixation errors by steering clear of the term ‘mechanical fall’, which might lead to a less thorough assessment of underlying intrinsic and external causes in older people, who are at high risk for future falls with associated high‐risk morbidity and mortality [45].

#### 2.2.4 Acopia and Social Admission

‘Acopia’ and ‘social admission’ are both ageist and pejorative terms, used to describe older people who have unmet care needs or are experiencing functional difficulties while implying they do not have any urgent medical reason for an ED visit or hospital admission. An older person who is admitted with a diagnosis of ‘acopia’ or ‘social admission’ often presents with multiple geriatric syndromes, multimorbidity and polypharmacy [46, 47, 48]. Furthermore, hospital mortality among older people with a diagnosis of social admission was alarmingly high at 34.9% [46] with one study finding it was associated with sepsis in 30%, and had a 22% mortality [48].

These terms suggest that the caregivers and/or the older person are the ‘problem’, which can hinder clinicians' critical thinking through anchoring bias and discourage exploration of the underlying causes.

We call for removing labels like ‘acopia’ or ‘social admission’ from our healthcare lexicon for older people who require hospital admission without an initial, clear, acute medical diagnosis. Instead, HCPs are encouraged to focus on describing presenting issues and the older person's comorbidities, degree of frailty, psychosocial context, and the caregiver's concerns rather than using pejorative labels.

#### 2.2.5 Ceiling of Care

The term ‘ceiling of care’ may imply that care and compassion cease if aggressive treatments are not the person's preferred options. Given that patients and families often fear abandonment on approaching end‐of‐life [49] and expressions of non‐abandonment correlate with higher family satisfaction [50], it is important that our communication is particularly sensitive to this fear and does not perpetuate it. Words alone are empty without accompanying actions. There should not be a ‘ceiling of care’ as care and compassion must be at the core of the management of all emergency patients.

We recommend the use of terms such as ‘goals of care’ or ‘care preferences’, as they do not risk implying that care and compassion will cease as end‐of‐life approaches. Even more important is provision of specific assurances that the patient will not be abandoned before death, and that all efforts will be made to ensure the patient's comfort [50].

A summary of the words and phrases discussed in this section that may lead to poor healthcare outcomes due to implicit bias, anchoring bias, premature diagnostic closure, or ageism is seen in Table 2.

**TABLE 2:** Words that may result in adverse healthcare outcomes through implicit bias, anchoring bias, premature diagnostic closure or ageism.

Age‐negative language	Age‐positive alternative
Poor historian	Assess for delirium or cognitive impairment; assess hearing and communication; where indicated, seek collateral history.
Pleasantly confused	Begin with descriptive terms to describe the behaviour, such as ‘patient lying in bed without agitation’ or ‘patient not verbally responding to questions’. Then use more precise language to describe the confusional state. For example: Acute confusion, ‘patient might have delirium’ Chronic confusion, ‘patient living with dementia’ Acute‐on‐chronic confusion, ‘patient experiencing delirium superimposed on dementia’
Mechanical fall	Use (Fall) as the diagnosis and describe the circumstances of the fall in the setting of extrinsic factors (e.g., contribution to fall of ill‐fitting shoes or slippery or uneven surface), or intrinsic factors (e.g., contribution to fall due to cardiogenic syncope), or fall with multifactorial extrinsic and intrinsic contributors
Acopia and social admission	Describe presenting issues, the older person's comorbidities, degree of frailty, psychosocial context and the caregiver's concerns
Ceiling of care	Use the term ‘*Goals of care*’ or ‘*Care preference*’

## 3 Conclusion

Words and language matter. Ageism exists. It is time to change the way we speak to and about older people and recognise that the language we use can directly and indirectly impact the patient experience and influence healthcare outcomes. Ageism can significantly influence the attitudes and actions (or inactions) of the community, the healthcare system, and government decision‐making bodies. Positive transformation begins with changing the words and language we employ in our everyday lexicon and all communications related to the care of older people, whether in research, academic writing, education or policy.

## Conflicts of Interest

The authors declare no conflicts of interest.

## Data Availability

Data sharing not applicable to this article as no datasets were generated or analysed during the current study.
